# AI-RiskX: An Explainable Deep Learning Approach for Identifying At-Risk Patients During Pandemics

**DOI:** 10.3390/bioengineering12101127

**Published:** 2025-10-21

**Authors:** Nada Zendaoui, Nardjes Bouchemal, Mohamed Rafik Aymene Berkani, Mounira Bouzahzah, Saad Harous, Naila Bouchemal

**Affiliations:** 1 Institute of Mathematics and Computer Science, Abdelhafid Boussouf University Center of Mila, Mila 43000, Algeria; n.bouchemal.dz@ieee.org (N.B.); m.bouzahzah@centre-univ-mila.dz (M.B.); 2 LIRE Laboratory of Constantine 2, Constantine 25000, Algeria; 3 LISI Laboratory of Intelligent Systems and Informatics, Mila 43000, Algeria; 4 LSEA Research Laboratory in Advanced Electronics Systems, University Yahia Fares of Medea, Medea 26000, Algeria; berkani.aymene@univ-medea.dz; 5 Department of Computer Science, College of Computing and Informatics, University of Sharjah, Sharjah P.O. Box 27272, United Arab Emirates; harous@sharjah.ac.ae; 6 LyRIDS ECE Ecole d’Ingénieurs de Paris, 75015 Paris, France; naila.bouchemal@ece.fr

**Keywords:** artificialintelligence, deep learning, explainable artificial intelligence, CNN, LSTM, decision making, at-risk patient

## Abstract

Pandemics place extraordinary pressure on healthcare systems, particularly in identifying and prioritizing high-risk groups such as the elderly, pregnant women, and individuals with chronic diseases. Existing Artificial Intelligence models often fall short, focusing on single diseases, lacking interpretability, and overlooking patient-specific vulnerabilities. To address these gaps, we propose an Explainable Deep Learning approach for identifying at-risk patients during pandemics (AI-RiskX). AI-RiskX performs risk classification of patients diagnosed with COVID-19 or related infections to support timely intervention and resource allocation. Unlike previous models, AI-RiskX integrates five public datasets (asthma, diabetes, heart, kidney, and thyroid), employs the Synthetic Minority Over-sampling Technique (SMOTE) for class balancing, and uses a hybrid convolutional neural network–long short-term memory model (CNN–LSTM) for robust disease classification. SHAP (SHapley Additive exPlanations) enables both individual and population-level interpretability, while a post-prediction rule-based module stratifies patients by age and pregnancy status. Achieving 98.78% accuracy, AI-RiskX offers a scalable, interpretable solution for equitable classification and decision support in public health emergencies.

## 1. Introduction

The COVID-19 pandemic has exposed critical weaknesses in healthcare systems, particularly in recognizing and prioritizing those at high risk. The elderly (≥60 years) showed the highest vulnerability, with severe outcomes reaching 85% [1,2], while children experienced up to 30% severe cases and 28% of Intensive Care Unit (ICU) admissions [3,4]. Pregnant women and patients with chronic conditions such as heart disease (52%) and kidney disease (48%) are also disproportionately affected [5], as summarized in Table 1. These risks stem from weakened immunity, underlying conditions, or physiological changes that reduce resilience to infection. Addressing such challenges requires intelligent, timely, and equitable risk stratification tools to support data-driven decision-making and optimize resource allocation during pandemics.

Artificial Intelligence has emerged as a valuable tool for medical classification and diagnostics; however, current models still exhibit notable limitations. First, most studies focus narrowly on a single disease [6,7], rarely accounting for coexisting health conditions or patient demographics [8]. Second, many existing models operate as “black boxes,” offering limited interpretability and lacking transparency in their decision-making processes [9,10]. Third, few systems address demographic-specific vulnerabilities—such as age or pregnancy—or provide interfaces suitable for real-time clinical deployment [9,10]. This lack of flexibility, transparency, and consideration of patient diversity hinders trust and the effective implementation of AI tools in real-world healthcare settings.

To address these multifaceted challenges, we propose AI-RiskX, a novel and interpretable deep learning framework for comprehensive multidisease risk assessment and stratification, supporting clinical decisions for patients with COVID-19 or related infections. First, AI-RiskX expands the modeling scope beyond single diseases by integrating five well-curated public datasets—covering heart disease, diabetes, asthma, chronic kidney disease, and thyroid disorders—into a unified learning framework. The datasets are harmonized and globally balanced using the Synthetic Minority Over-sampling Technique (SMOTE) [11,12], ensuring unbiased learning and meaningful stratification across diverse conditions. Second, the framework enhances interpretability through explainable AI components that reveal how individual clinical features influence predictions. This allows clinicians to trace and understand the reasoning behind model outputs, fostering transparency and trust in AI-driven decision-making. Third, AI-RiskX incorporates demographic- and context-aware mechanisms that reflect real patient diversity considering factors such as age, comorbidities, or pregnancy and its modular design supports smooth integration into real-time clinical workflows.

AI-RiskX combines several innovations within a unified architecture: (i) a harmonized multidisease dataset supporting unbiased training; (ii) a hybrid convolutional neural network–long short-term memory (CNN–LSTM) backbone for robust multi-label prediction; (iii) a demographic-aware rule-based module for refined classification; and (iv) SHapley Additive exPlanations (SHAP) for local and global interpretability, dynamically adapting predictions with crucial demographic factors such as age and pregnancy status.

The core contributions of this study are as follows:

- **Unified dataset:** Integrating five well-curated public datasets covering heart disease, diabetes, asthma, chronic kidney disease, and thyroid disorders (Table 1), AI-RiskX focuses on clinically actionable features such as blood tests, vital signs, and comorbidities. These conditions were selected for their high prevalence, frequent co-occurrence in vulnerable populations, and links to adverse pandemic outcomes. The harmonized dataset mitigates class imbalance via SMOTE and is supported by epidemiological evidence and WHO guidance [13], ensuring robust, generalizable multidisease learning [14].
- **Hybrid CNN–LSTM model:** Employing a parallel convolutional–recurrent design that captures both spatial patterns and temporal dependencies in structured clinical data, thereby enhancing prediction robustness and accuracy over conventional pipelines.
- **Demographic-aware rule-based:** Incorporating a lightweight, rule-based classification module that integrates clinically significant demographic factors—such as advanced age (≥60 years) and pregnancy status, as outlined in Table 1—to refine patient classification into three risk categories: high risk, at risk, and not at risk.
- **Explainability:** Embedding SHapley Additive exPlanations (SHAP) to deliver transparent local and global interpretability, enabling clinicians to understand the contribution of each feature to model predictions and foster trust in AI-assisted decision-making.

The remainder of this paper is structured as follows: Section 2 reviews related studies, Section 3 details the proposed methodology, Section 4 presents the results and analysis, and Section 5 concludes with key findings and future directions.

## 2. Related Work

Artificial Intelligence (AI) has significantly advanced healthcare by enabling early diagnosis, risk stratification, and clinical decision-making support. Despite these advancements, most AI models struggle with real-world applicability owing to their narrow disease focus, limited interpretability, and poor sensitivity to patient diversity.

Our study focuses on risk classification for patients already diagnosed with COVID-19. The proposed AI-RiskX framework evaluates disease severity and multimorbidity risk profiles by leveraging structured clinical data, comorbidities, and demographic information. Unlike models developed for infection detection [15,16], AI-RiskX emphasizes patient stratification and clinical decision support. Previous research highlights two major limitations in existing approaches: (1) a focus on single-disease classification using fragmented datasets, and (2) deep learning architectures with limited interpretability. These shortcomings motivated the development of AI-RiskX, a unified and explainable system designed to address the complex challenges of pandemic healthcare.

### 2.1. Single-Disease Focus and Dataset Fragmentation

Most existing AI models in healthcare predict a single disease using isolated datasets, limiting their applicability in real clinical settings with multiple chronic conditions or overlapping symptoms. We selected studies that used machine learning or deep learning for disease prediction, incorporated explainability methods such as SHAP or Local Interpretable Model-Agnostic Explanations (LIME), and relied on structured, reproducible datasets; studies lacking interpretability or based solely on imaging or proprietary data were excluded to align with AI-RiskX.

El-Sofany et al. [17] built a diabetes prediction system using XGBoost and SMOTE, achieving an accuracy of 97.4%. However, their approach was confined to one disease and lacked integration of explainability or demographic profiling. Similarly, Guleria et al. [18] developed a cardiovascular disease model using SVM with SHAP and LIME but trained it on a small dataset, missing deep learning capabilities.

Yilmaz et al. [19] applied Random Forest, Logistic Regression, and SVM to predict coronary heart disease and achieved 92.9% accuracy. However, their model did not incorporate interpretability tools or address the needs of the vulnerable subpopulations. Prathibha et al. [20] and Zhang et al. [21] implemented CNNs such as ResNet and Xception for thyroid disorder detection using ultrasound images with high accuracy (97%); however, their models relied solely on imaging data and lacked multimodal integration.

In the context of asthma detection, Awal et al. [22] introduced a Bayesian Optimization-based model (BOMLA), whereas Heris et al. [23] utilized KNN with Relief-F. Both faced challenges owing to small datasets and limited generalizability.

During the COVID-19 pandemic, Bottrighi et al. [24] and Quiroz et al. [25] developed models focused on deterioration prediction using rule-based and neural network methods, respectively. Although beneficial, these models were not adaptable beyond COVID-19-specific data.

Ahmed et al. [26] applied genomic data and statistical modeling for cardiovascular risk prediction but omitted critical clinical features such as comorbidities, symptoms, or demographics, limiting their real-world clinical value.

Most recent studies continue to focus on single-disease modeling. For example, Talukder et al. [27] proposed HXAI-ML, a hybrid explainable model for cardiovascular heart disease combining SHAP, LIME, and permutation importance, achieving over 98% accuracy on the Cleveland and Framingham datasets. Similarly, Bilal et al. [28] introduced an XAI-driven system for cardiovascular forecasting using large-scale EMR data (≈308,000 samples), enhancing transparency but remaining disease-specific. In the respiratory domain, Mahmood et al. [29] applied AutoML with explainable AI for asthma outcome prediction, while Lyu et al. [30] classified asthma severity using vocal biomarkers and SHAP analysis. Despite their robustness, these models remain limited to single-disease contexts without integration across comorbidities or demographic factors.

Collectively, these studies reveal a fragmented landscape: models are overly disease-specific, datasets are isolated, and key clinical variables, such as comorbidities and demographics, are frequently overlooked. This fragmentation underscores the necessity of using unified multiple diseases data.

### 2.2. Deep Learning and Explainability

Deep learning (DL), particularly Convolutional Neural Networks (CNNs), have shown strong performance in healthcare for image classification and structured data analysis. Their ability to extract complex patterns makes them ideal for use in diagnostic tasks. For example, Srinivasu et al. [31] developed a CNN–Bi-LSTM model for diabetes monitoring with 96.44% accuracy, and Prathibha et al. [20] utilized a ResNet model to classify thyroid conditions from ultrasound images with 97% accuracy.

Although AI models have achieved remarkable predictive accuracy, many still operate as “black boxes,” offering limited transparency regarding how decisions are made. Recent advances in explainable AI (XAI) have sought to address this issue, particularly in COVID-19 prognosis studies. For instance, Chadaga et al. [32] and Ozawa et al. [33] proposed interpretable models to enhance clinical trust and understanding.

Post hoc explanation techniques such as SHAP (SHapley Additive exPlanations) and LIME (Local Interpretable Model-Agnostic Explanations) have further advanced model interpretability. SHAP, in particular, offers a strong theoretical foundation for both local and global feature attribution [34]. For example, Duckworth et al. [35] used SHAP to monitor data drift in emergency care, and Zhao et al. [36] applied it to immune disorder prediction. Similarly, Tasin et al. [37] compared LIME and SHAP in diabetes modeling and found SHAP to provide superior clinical interpretability.

### 2.3. Addressing Gaps with AI-RiskX

To address these persistent limitations, we introduce AI-RiskX, an explainable multidisease prediction approach tailored for real-world healthcare and pandemic response. First, to overcome the single-disease focus of most previous studies, AI-RiskX supports risk prediction across five prevalent chronic diseases, diabetes, heart disease, asthma, kidney disease, and thyroid disorders, using a harmonized and balanced dataset. Second, to capture both spatial and temporal dependencies in clinical data, a hybrid CNN–LSTM architecture is employed, with SMOTE-based preprocessing applied to mitigate class imbalance. Third, to resolve the lack of demographic awareness and improve usability, a rule-based module profiles vulnerable populations (the elderly, pregnant women, and children). Finally, to address the opacity of deep models, AI-RiskX embeds SHAP within the approach, providing transparent case-level and population-level explanations that enhance clinical interpretability. A comparative overview of these prior models and the distinguishing features of AI-RiskX is summarized in Table 2.

## 3. Methodology

This section outlines the methodology of the proposed AI-RiskX approach, an explainable system developed to support the early detection and risk classification of vulnerable patients during pandemics. Building upon our previous work on AI-LMS (AI-Based Long-Term Monitoring) [38], which focused on continuous patient monitoring using IoMT and machine learning, AI-RiskX is introduced as a dedicated subsystem specifically designed for risk stratification. In AI-LMS, IoMT sensors, mobile cloud computing, and AI models were combined to enable long-term monitoring and alert generation for at-risk patients during pandemics such as COVID-19. AI-RiskX refines this framework by isolating the risk evaluation phase, focusing solely on the identification and classification of patients according to their vulnerability level based on comorbidities, demographic attributes, and clinical indicators. This targeted component serves as the decision-support core of AI-LMS, providing explainable, data-driven risk assessment before continuous monitoring takes place.

AI-RiskX is designed as a four-phase approach that integrates data processing, disease prediction, demographic-aware evaluation, and explainability to deliver clinically meaningful insights.

**Phase I: Data Processing.** Heterogeneous datasets covering five major chronic conditions ( diabetes, heart disease, asthma, chronic kidney disease, and thyroid disorders) were harmonized into a unified multidisease dataset. Unlike prior studies that relied on single-disease data, this unified construction process improves generalizability across multiple conditions. Standard preprocessing, including normalization and SMOTE, was applied to ensure balanced class distributions.

**Phase II: Patient Risk Assessment Model.** The core of this phase is a hybrid CNN–LSTM architecture designed to extract multiscale spatial features (capturing correlations among clinical variables) and long-term temporal dependencies (reflecting sequential relationships in patient profiles), generating robust representations for classification across multiple disease classes. By combining spatial and temporal learning, this phase enhances the predictive performance of structured health data compared to conventional single-stream models.

**Phase III: Demographic-Aware Risk Evaluation.** A rule-based module identifies vulnerable groups, such as the elderly and pregnant women, and integrates this demographic information with disease predictions to stratify patients into three categories: high risk, at risk, and not at risk. This integration ensures fairness, ethical decision support, and targeted interventions, addressing the limitations of prior models that largely overlook demographic factors.

**Phase IV: Explainability.** To address the opacity of deep learning models, Explainable Artificial Intelligence (XAI) techniques, specifically SHAP, are embedded to provide transparent explanations at both individual and population levels. By linking prediction outcomes to clinical features, this phase strengthens interpretability, trust, and clinical acceptance, distinguishing AI-RiskX from the traditional black-box systems.

**Figure 1:**
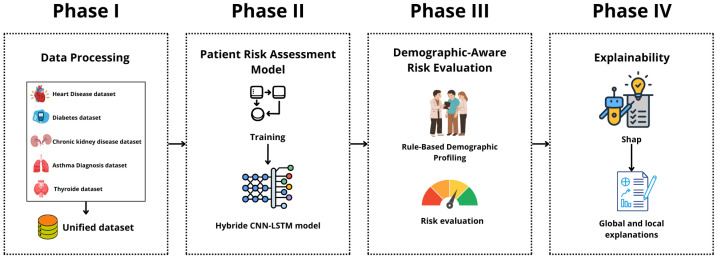
AI-RisX phases.

As the identification core of the AI-LMS approach, AI-RiskX refines the early-stage decision-making component by offering an explainable, data-driven evaluation of high-risk patients across multiple chronic conditions. The complete workflow is illustrated in Figure 1 The following subsections describe each component in detail.

### 3.1. Data Processing

This section outlines the data foundation of the proposed AI-RiskX system. It begins with a description of the datasets used for model training and evaluation, followed by the preprocessing and harmonization steps implemented to standardize, balance, and prepare the data for deep learning analysis. These processes ensure input consistency and robustness across heterogeneous medical sources.

#### 3.1.1. Dataset Description

Unlike earlier studies focusing on single-disease prediction, our work leverages five distinct medical datasets to train and evaluate the AI-RiskX framework. These datasets cover heart disease, diabetes, thyroid disorders (hypo/hyperthyroidism), asthma, and chronic kidney disease (CKD). Each includes both demographic and clinical attributes relevant to disease-specific diagnostics, providing a comprehensive basis for patient-level risk assessment. For the purpose of this study, we assume that all patients are affected by a pandemic scenario (e.g., COVID-19), so the model assesses vulnerability based on pre-existing conditions.

The UCI Heart Disease dataset [39] provides structured cardiac health records and is commonly used for binary classification of heart conditions. The HealthCare Diabetes dataset [40] comprises 768 patient instances with standard clinical features and binary outcome labels. Thyroid dysfunction detection is based on the Hypo/Hyperthyroidism dataset [41], containing mixed numerical and categorical features for precise classification. The Asthma Diagnosis dataset [42] includes anonymized patient-level records with diverse attributes suitable for supervised asthma identification. Finally, CKD prediction employs the Chronic Kidney Disease dataset [43], consisting of 400 labeled samples for disease presence or absence.

Together, these heterogeneous data sources form a unified multidisease foundation for robust prediction and risk stratification. The preprocessing and harmonization pipeline described below integrates these datasets into a cohesive, balanced, and clinically interpretable dataset—AI-RiskX.

#### 3.1.2. Data Preprocessing and Harmonization

**Figure 2:**
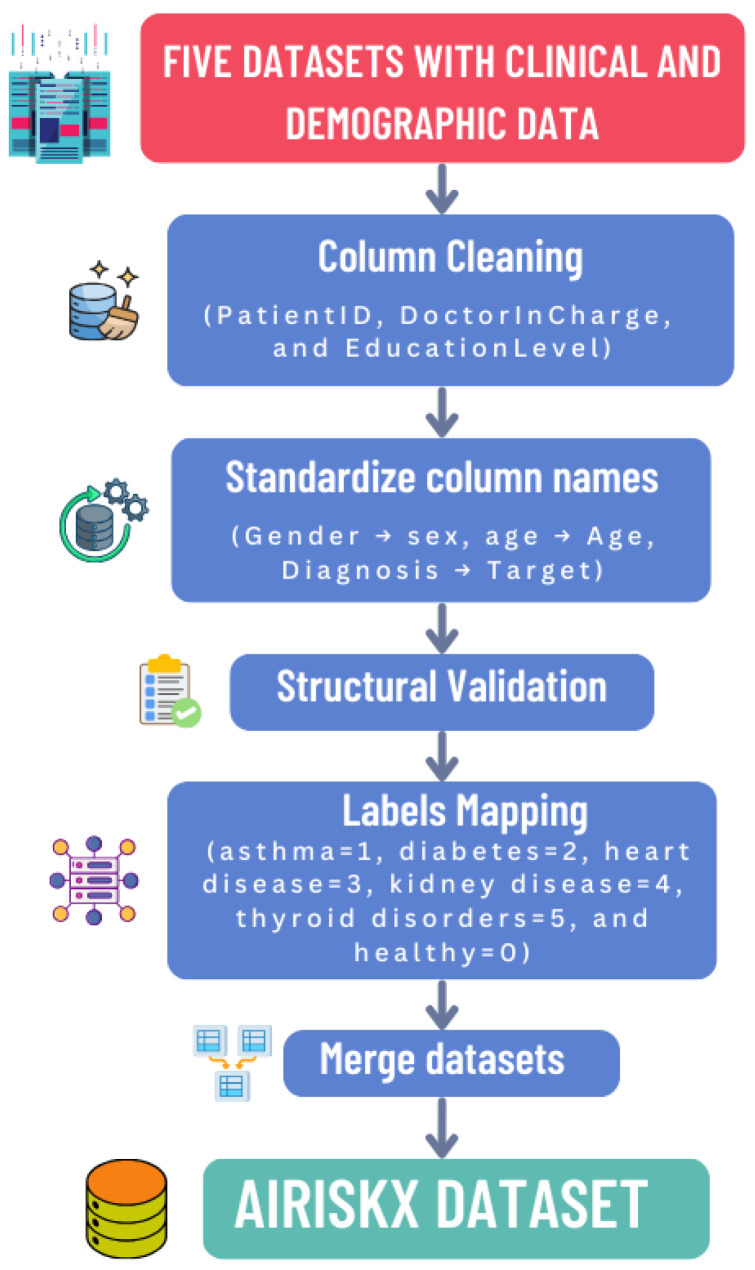
AI-RiskX dataset creation and harmonization workflow.

Normalization and harmonization were essential to ensure structural, semantic, and statistical consistency across the five heterogeneous datasets. The complete workflow is summarized in Figure 2.

To integrate datasets with distinct structures and variable names, we developed a harmonization pipeline comprising the following steps to ensure structural and semantic consistency across the five datasets:

- **Column Cleaning:** Non-informative or administrative attributes such as PatientID, DoctorInCharge, and EducationLevel were removed to eliminate redundancy and potential privacy risks.
- **Schema Alignment:** Attribute names were standardized across datasets (e.g., Gender → sex, Diagnosis → Target) to ensure consistency and prevent schema conflicts during merging.
- **Structural Validation:** Each dataset was verified to contain the same number of features (79) with consistent data types (numeric, categorical, or binary), guaranteeing interoperability and preventing mismatches.
- **Label Mapping:** Diagnostic labels were harmonized into a unified multi-class structure: healthy (0), asthma (1), diabetes (2), heart disease (3), chronic kidney disease (4), and thyroid disorder (5). This unified labeling enables multidisease classification within a single framework.
- **Handling Missing Features:** Some features were missing in certain datasets. For each missing value, we first checked if a similar or proxy feature existed that could represent the same clinical concept; if found, it was mapped to the unified feature. If no comparable feature was available, simple imputation was applied: mean imputation for continuous features and mode imputation for categorical features. This ensured that all 79 features were consistently present across datasets, enabling robust multidisease modeling. This approach preserves feature completeness, maintains structural consistency, and is computationally efficient.

The harmonized dataset comprises 26,125 samples and 79 unified features, reshaped into a two-dimensional structure of size (79,1), where each instance corresponds to a patient record and its target class. Table 3 summarizes the dataset composition, while Table 4 categorizes features into thematic groups spanning demographics, lifestyle, and disease-specific indicators.

Following harmonization, the five disease datasets were merged to form the unified AI-RiskX dataset. Due to strong class imbalance—particularly for asthma with only 124 samples—the Synthetic Minority Oversampling Technique (SMOTE) was employed to balance the classes. Rather than oversampling each dataset separately, SMOTE was applied globally to the integrated dataset, ensuring uniform class distribution and improving model generalizability.

SMOTE was applied only to training data to avoid leakage. For small datasets (e.g., asthma), oversampling was limited, and statistical checks confirmed consistency with original data. Harmonization kept clinically relevant shared features for coherent multidisease learning. Each new sample $x_{\mathrm{new}}$ is created based on an existing minority instance *x* and one of its *k* nearest neighbors $x_{\mathrm{nn}}$ using a random scalar λ∈[0,1], as defined in Equation (1):

(1) $$x_{\mathrm{new}}=x+\lambda \cdot \left(x_{\mathrm{nn}}-x\right)$$

The Euclidean distance between samples, computed in Equation (2), is used to identify nearest neighbors:

(2) $$d\left(x_{i},x_{j}\right)=\sqrt{\sum_{m=1}^{M}{\left(x_{i,m}-x_{j,m}\right)}^{2}}$$

SMOTE minimizes the total distance across minority instances as shown in Equation (3):

(3) $$\min\sum_{i=1}^{N_{m}}\sum_{j=1}^{k}d\left(x_{i},x_{i,j}\right)$$

Table 5 presents the class distribution before and after balancing, demonstrating how SMOTE corrects extreme disparities among disease categories.

For normalization, Min–Max scaling was applied to rescale all features within the [0,1] range:

(4) $$x_{N}=\frac{x-\min(x)}{\max(x)-\min(x)}$$

This transformation facilitates faster convergence during training and reduces numerical instability. After normalization, the dataset was reshaped into a two-dimensional structure suitable for CNN-based feature extraction.

For dataset partitioning, the normalized data were randomly shuffled and divided into three distinct subsets for model development and evaluation. Specifically, 70% of the data were used for training to enable model learning and parameter optimization, 20% were allocated for validation to fine-tune hyperparameters and monitor performance during training, and the remaining 10% were reserved as an independent testing set to provide an unbiased assessment of the model’s generalization capability.

### 3.2. Patient Risk Assessment Model

The proposed AI-RiskX framework was developed for accurate multidisease classification and interpretable patient risk assessment using structured clinical data such as vital signs, laboratory results, and diagnostic indicators. At its core, the model employs a hybrid CNN–LSTM architecture designed to capture both localized feature interactions and long-range dependencies within patient profiles.

Before introducing the deep learning architectures, three classical machine learning baselines were implemented to establish performance references on structured clinical data:

- **Logistic Regression:** A simple linear classifier was implemented as a baseline. Input features were standardized using StandardScaler, and the model was trained with L2 regularization (C = 1.0) using the lbfgs solver for up to 1000 iterations. Stronger regularization slightly decreased recall, but this configuration provided a stable and interpretable benchmark.
- **Random Forest:** A Random Forest ensemble with 200 trees (n_estimators = 200) was trained using Gini impurity and balanced class weights to address class imbalance. Bootstrap aggregation (bootstrap = True) improved robustness, and hyperparameters were optimized via 5-fold cross-validation to maximize the macro F1-score. This model captures nonlinear feature interactions and provides interpretable variable importance.
- **XGBoost:** An XGBoost classifier was configured with learning_rate = 0.1, max_depth = 6, and n_estimators = 200. The model used early stopping (patience = 10) with eval_metric = ‘logloss’, L2 regularization (lambda = 1), and subsampling (subsample = 0.8) to improve generalization. Combined with SMOTE-based oversampling, XGBoost effectively handled complex feature interactions and class imbalance.

Building upon these baselines, the proposed CNN–LSTM architecture was benchmarked against representative deep learning models commonly used in medical AI, including CNN [44], LSTM [45], BiLSTM [46], and a transformer-based LLM–BERT model [47]:

- **Convolutional Baseline (CNN):** A single-stream 1D CNN was implemented to capture spatial dependencies among the 79 clinical features. The network consisted of two Conv1D layers (64 filters; kernel size = 3), followed by BatchNormalization, MaxPooling1D, and two dense layers (64 and 32 neurons) with ReLU activation. Dropout (0.4) was applied for regularization. The model was trained using the Adam optimizer (lr = 0.001), batch size = 32, and 100 epochs with early stopping (patience = 10). This baseline evaluates the effectiveness of purely convolutional feature extraction without sequential modeling.
- **Recurrent Baselines (LSTM and BiLSTM):** To model sequential dependencies among input features, LSTM-based architectures were evaluated. The standard LSTM network employed two stacked layers (64 and 32 units) with dropout (0.3 and 0.4) to prevent overfitting, trained for up to 120 epochs using Adam (lr = 0.001) and early stopping (patience = 10). The bidirectional LSTM (BiLSTM) [46] extended this by processing sequences in both forward and backward directions to capture bidirectional dependencies. It comprised two BiLSTM layers (128 units each) with dropout rates of 0.2 and 0.4, followed by a dense softmax classifier. Training continued for 150 epochs with early stopping and L2 regularization (lambda = 0.001) for better generalization. These recurrent architectures assess the ability to learn temporal and contextual feature relationships.
- **Transformer-Based Baseline (LLM–BERT):** To investigate the applicability of large language models to structured clinical data, an adapted BERT model was fine-tuned for six-class classification (five diseases and one healthy control). Each patient record was transformed into a text-like representation of feature–value pairs (e.g., “Age: 45; Gender: Male; BloodPressure: High; Glucose: Normal; …”), allowing the tokenizer to embed clinical variables contextually. The tokenized inputs were processed by a pre-trained BERT model [47], fine-tuned for 10 epochs using AdamW (lr = 2 × 10^−5^) with linear warmup and dropout (0.1) on the classifier head. This model serves as a proof of concept for adapting LLMs to tabular medical data.
- **Proposed CNN–LSTM Hybrid Architecture:** The core AI-RiskX model integrates Convolutional Neural Networks (CNNs) and Long Short-Term Memory (LSTM) networks in a parallel hybrid configuration to exploit both spatial and sequential dependencies in clinical features. CNNs efficiently capture localized correlations and hierarchical feature patterns, while LSTMs model sequential dependencies that may reflect implicit clinical relationships among patient variables [44,45,48].

**Figure 3:**
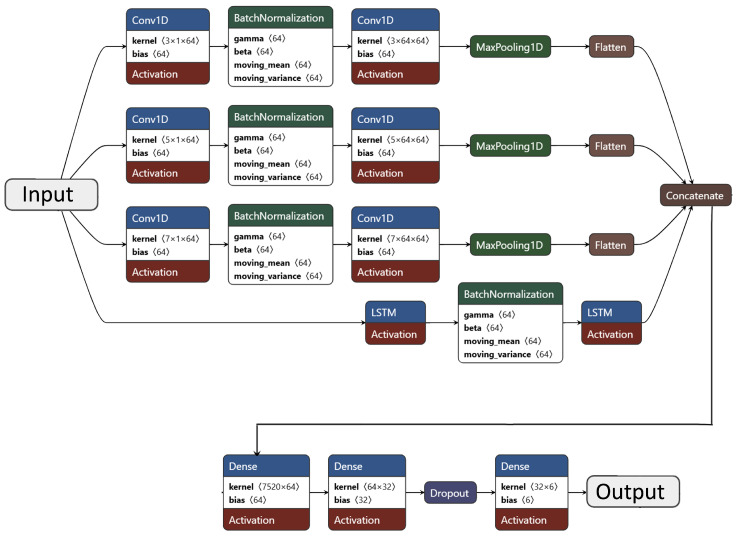
Proposed AI-RiskX architecture (CNN–LSTM). The model fuses three convolutional branches and one LSTM branch to capture multi-resolution spatial and sequential dependencies in parallel.

The architecture comprises three parallel CNN branches (kernel sizes 3, 5, and 7) and one LSTM branch. The convolutional branches each include two Conv1D layers (64 filters; ReLU activation), BatchNormalization, and MaxPooling1D (pool size = 2). Kernel sizes of 3, 5, and 7 enable multi-resolution feature extraction. The LSTM branch consists of two stacked LSTM layers (64 and 32 units) with batch normalization to ensure stable learning. The outputs from all branches are flattened and concatenated, followed by two dense layers (64 and 32 neurons; ReLU activation) and a dropout layer (rate = 0.3). The final softmax layer produces the probability distribution across six classes—five chronic diseases and one healthy control. A detailed layer-wise summary of this architecture is presented in Table 6, and its structural design is illustrated in Figure 3.

Model training used categorical cross-entropy loss with a 70–20–10 train–validation–test split. All data were normalized, and SMOTE-based balancing was applied only to training sets. A stratified 5-fold cross-validation procedure further evaluated generalization, ensuring balanced class representation across folds. Each fold was trained for 30 epochs with early stopping, and performance was averaged across folds. Evaluation metrics included accuracy, precision, recall, F1-score, and AUC, as detailed in Section 4.

Overall, the hybrid CNN–LSTM architecture demonstrated superior robustness and interpretability by combining spatial and sequential learning in a unified framework, enabling AI-RiskX to model complex clinical dependencies and support patient risk classification.

### 3.3. Demographic-Aware Risk Evaluation

The AI-RiskX approach includes a post-classification layer for demographic-aware risk profiling. This layer performs three functions—age group classification, pregnancy status identification, and composite patient risk assessment while operating alongside the disease prediction model without affecting its training or classification. The overall logic is summarized in Algorithm 1.

Patients are first categorized into clinically relevant age groups to capture age-related vulnerability: children (<18 years), adults (18–59 years), and elderly (≥60 years). Modeling age as a categorical attribute introduces medically interpretable logic that complements numerical features and enhances risk contextualization.

Pregnancy status is included as a binary attribute to account for gender-specific vulnerabilities, ensuring that this critical demographic group is appropriately considered.

Finally, the module combines disease predictions with demographic indicators to produce three risk levels: high risk (diseased and vulnerable), at risk (diseased but not vulnerable), and not at risk (no disease and not vulnerable).
**Algorithm 1** Check Patient Risk**Input:**data, model**Output:** Patient risk assessmentExtract age_group and pregnancy_status from dataPredict disease using model and select the category with the highest probabilityDefine high-risk criteria: age_group (child or elderly) or pregnancy_status (pregnant)**If** predicted disease ≠ Healthy:
(a)**If** patient meets high-risk criteria: HIGH RISK: predicted disease and risk factors(b)**Else:** AT RISK: predicted disease
**ElseIf** patient meets high-risk criteria: AT RISK due to high-risk condition**Else:** NOT at Risk

### 3.4. Explainability

AI-RiskX uses SHAP (SHapley Additive exPlanations) to interpret model predictions. SHAP provides global and local explanations based on cooperative game theory, ensuring local accuracy and capturing feature interactions. Recent studies have demonstrated SHAP’s effectiveness in highlighting clinically relevant predictors, such as age and disease stage in cancer prognosis [49], and its robustness when applied to deep learning models in healthcare [50]. These findings support SHAP’s reliability and reproducibility in clinical environments [51].

The Shapley value for a model *f* and input $x\in R^{M}$ with *M* features is defined as follows [34]:

(5) $$\phi_{i}\left(f,x\right)=\sum_{S\subseteq F\setminus \{i\}}\frac{|S|!(M-|S|-1)!}{M!}\left[f_{S\cup \{i\}}\left(x_{S\cup \{i\}}\right)-f_{S}\left(x_{S}\right)\right]$$

where *F* is the set of all features, *S* is a subset excluding *i*, and $f_{S}\left(x_{S}\right)$ is the expected model output using only features in *S*. Key properties— additivity, local accuracy, and consistency—ensure reliable feature attribution. Approximation techniques such as Deep SHAP are used for computational efficiency in large networks [34].

Within AI-RiskX, SHAP is applied post hoc to the fused CNN–LSTM feature representation before the final classification layer, capturing both spatial and sequential dependencies. SHAP interprets structured clinical features, while the demographic-aware rule-based module applies explicit clinical logic (e.g., age and pregnancy), providing complementary interpretability through quantitative and rule-based transparency.

## 4. Results

This section presents the experimental evaluation of the proposed AI-RiskX system, its comparative performance against both classical machine learning and deep learning baselines, and details of its practical implementation.

### 4.1. Overall Performance Evaluation

To evaluate the necessity and effectiveness of deep learning techniques, several classical machine learning models (Logistic Regression, Random Forest, and XGBoost) were first trained and tested on both the combined and combined–balanced datasets. Their performance results are summarized in Table 7.

On the combined dataset, Logistic Regression achieved an accuracy of 89.95%, while Random Forest and XGBoost reached 96.77% and 96.99%, respectively. When trained on the combined–balanced dataset, the performance of Logistic Regression decreased to 70.68%, whereas Random Forest and XGBoost maintained strong results with accuracies of 96.84% and 96.98%. These findings suggest that although traditional machine learning algorithms can perform competitively, they struggle to capture the complex, nonlinear relationships and multidisease dependencies inherent in heterogeneous healthcare data—especially under class imbalance conditions.

To further assess model robustness, the proposed hybrid CNN–LSTM model was compared with various deep learning architectures, including CNN, LSTM, BiLSTM, and LLM-BERT, as also presented in Table 7. On the combined–balanced dataset, the hybrid CNN–LSTM achieved the highest accuracy of 98.78%, outperforming CNN (98.67%), LSTM (96.97%), BiLSTM (96.13%), and LLM-BERT (86.02%). Similarly, on the combined dataset, CNN–LSTM maintained superior performance with 94.55% accuracy.

When evaluated on individual disease datasets, BiLSTM exhibited the best performance for heart disease, diabetes, HHT, and CKD, while LSTM achieved the highest accuracy for AD. These results demonstrate that integrating CNN’s spatial feature extraction with LSTM’s temporal learning capabilities significantly enhances the system’s ability to model inter-disease relationships and capture complex clinical patterns, leading to improved generalization across multiple health conditions.

### 4.2. Ablation Study

To better understand the contribution of each component within the proposed architecture, we conducted an ablation study on the balanced dataset. The model variants were as follows: the CNN-only model, which serves as a spatial feature extractor without sequential modeling; the LSTM-only model, which functions as a sequential model without convolutional representation; and the CNN–LSTM (proposed) model, which combines both convolutional and temporal layers.

As shown in Table 8, excluding either the CNN or LSTM component resulted in reduced predictive performance. The CNN-only model achieved an accuracy of 98.67%, while the LSTM-only model reached 96.97%. In contrast, the full hybrid CNN–LSTM achieved the best overall performance, with 98.78% accuracy, 98.79% precision, 98.77% recall, and an F1-score of 98.77%.

These results highlight the complementary strengths of both modules: CNN layers effectively capture spatial correlations among clinical features, while LSTM layers model sequential dependencies across patients’ health records. Together, they provide a balanced representation that enhances the accuracy and robustness of multidisease risk classification across heterogeneous clinical datasets.

### 4.3. Cross-Validation Performance

The results of the stratified 5-fold cross-validation are summarized in Table 9. These results represent the average model performance and its variability across the five folds. The proposed hybrid CNN–LSTM model demonstrated highly consistent outcomes, achieving an average accuracy of 98.72% and an AUC of 99.52%. The low standard deviations observed across all metrics indicate that the model performs stably and is not overly dependent on a particular data partition.

To further validate the robustness of the proposed AI-RiskX model, a statistical significance analysis was conducted using the approximate randomization test [52] and non-parametric evaluation procedures [53,54], following standard practices in machine learning assessment. Considerations for mitigating evaluation biases in imbalanced datasets were also incorporated [55]. The reliability of the performance metrics was assessed using paired *t*-tests across the five stratified folds, with 95% confidence intervals for each metric *M* computed as follows:

(6) $$CI_{95\%}=\bar{M}\pm t_{\alpha /2,k-1}\times \frac{s}{\sqrt{k}}$$

where $\bar{M}$ is the mean performance, *s* is the standard deviation, and *k* is the number of folds. For instance, with an accuracy of 98.72±0.11, the corresponding 95% confidence interval is [98.58,98.86].

To assess whether the performance improvements were statistically significant, paired *t*-tests were conducted between the hybrid CNN–LSTM and the baseline models, using

(7) $$t=\frac{\bar{d}}{s_{d}/\sqrt{k}}$$

where $d_{i}$ denotes the difference in performance between the CNN–LSTM and each baseline model for fold *i*. All obtained *p*-values were below 0.05, confirming that the observed gains are statistically significant and unlikely to result from random variation.

### 4.4. ROC Curve

**Figure 4:**
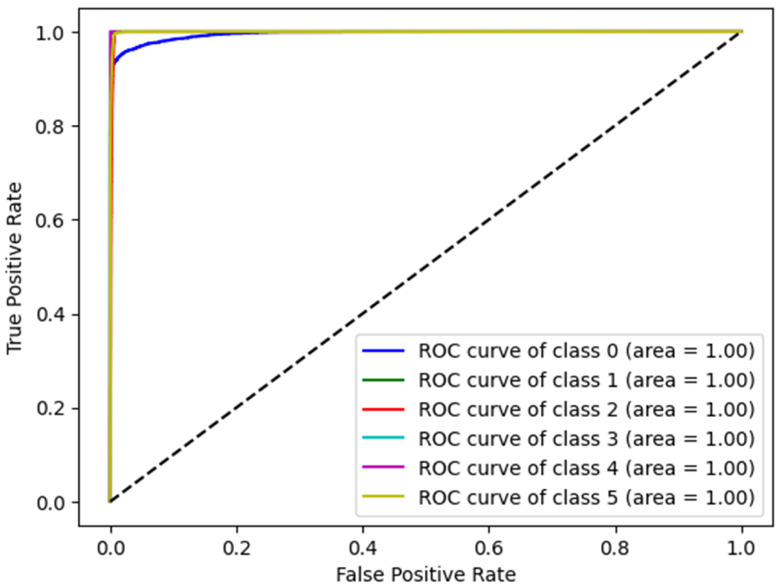
ROC curves of the CNN–LSTM model for multidisease classification on AI-RiskX.

Receiver Operating Characteristic (ROC) curves and Area Under the Curve (AUC) were used to evaluate the overall effectiveness of the proposed model. The ROC curve plots the True Positive Rate (TPR) against the False Positive Rate (FPR) across various thresholds, thereby providing a comprehensive visualization of the model’s performance. Figure 4 presents the ROC curves of the proposed model corresponding to different disease categories, demonstrating excellent classification performance in all categories, with curves close to the upper right corner. The AUC for each class was at an optimal rate of 1.00, indicating perfect accuracy and an exceptional discriminative capability. These results validate the robustness of the proposed model for disease detection.

### 4.5. Confusion Matrix

**Figure 5:**
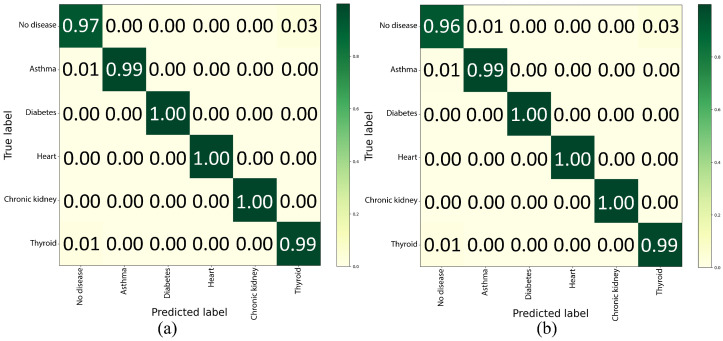
Confusion matrix of the CNN–LSTM model on AI-RiskX: (**a**) validation set and (**b**) testing set results.

Figure 5 illustrates the confusion matrix (CM) of the proposed model. It provides a detailed evaluation of the classification performance for various diseases. The results of the CM validation (Figure 5a) demonstrate a high classification accuracy, as indicated by the strong diagonal in the CM. Most instances were correctly classified; however, minor misclassifications were observed. Specifically, 3% of patients without disease were misclassified as having thyroid disease, while 1% of diabetes cases were incorrectly labeled as no disease. Similarly, 1% of thyroid cases were misclassified as no disease. These misclassifications likely arise from the overlapping feature representations between no disease and thyroid disease, as well as between no disease and diabetes. These results confirm the robustness of the proposed CNN-LSTM model for the accurate detection of multiple diseases in patients. The high classification performance, with diagonal performance ranging from 97% to 100%, highlights the reliability of the model, whereas the relatively minor off-diagonal misclassifications indicate potential areas for further refinement and optimization.

As shown in Figure 5b, the CM for the testing and generalization phase confirms the validation outcomes. Specifically, the model accurately identified most diseases, exhibiting a performance range between 96% and 100%, with only minor misclassifications quantified as follows: A total of 4% of instances from no disease were misclassified specifically, 1% as diabetes and 3% as thyroid. Additionally, 1% of the diabetes cases were misidentified as having no disease. Furthermore, 1% of thyroid instances were incorrectly categorized as no disease. These misclassifications arise from similarities and variability among the classes. These results demonstrate the potential of the AI-RiskX system for health monitoring and disease detection.

### 4.6. Explainability with SHAP

Model transparency is essential for clinical adoption in high-stakes healthcare AI systems. To address this, SHAP (SHapley Additive Explanations) was integrated into the AI-RiskX pipeline as a post hoc interpretability tool. After training the complete CNN–LSTM model, SHAP was applied to the fused feature vector and output of the concatenated convolutional and LSTM branches—prior to the dense classification layers. This enables the interpretation of the joint feature representation, thereby capturing the combined influence of both local and sequential dependencies.

SHAP was applied in primary data configurations:

- **Individual disease datasets:** SHAP was used to interpret predictions from the AI-RiskX model trained and evaluated separately for each disease-specific dataset. This helped to identify the key features associated with each condition.
- **Combined multidisease dataset:** SHAP was applied to the fused representation in the unified model trained on the merged dataset, to assess whether clinically relevant signals were preserved across diseases despite dataset integration.

SHAP provided both global and local explanations:

- **Global SHAP values** identify the most influential features across the entire dataset.
- **Local SHAP values** explain individual predictions by quantifying the contribution of each feature to a specific case.

**Figure 6:**
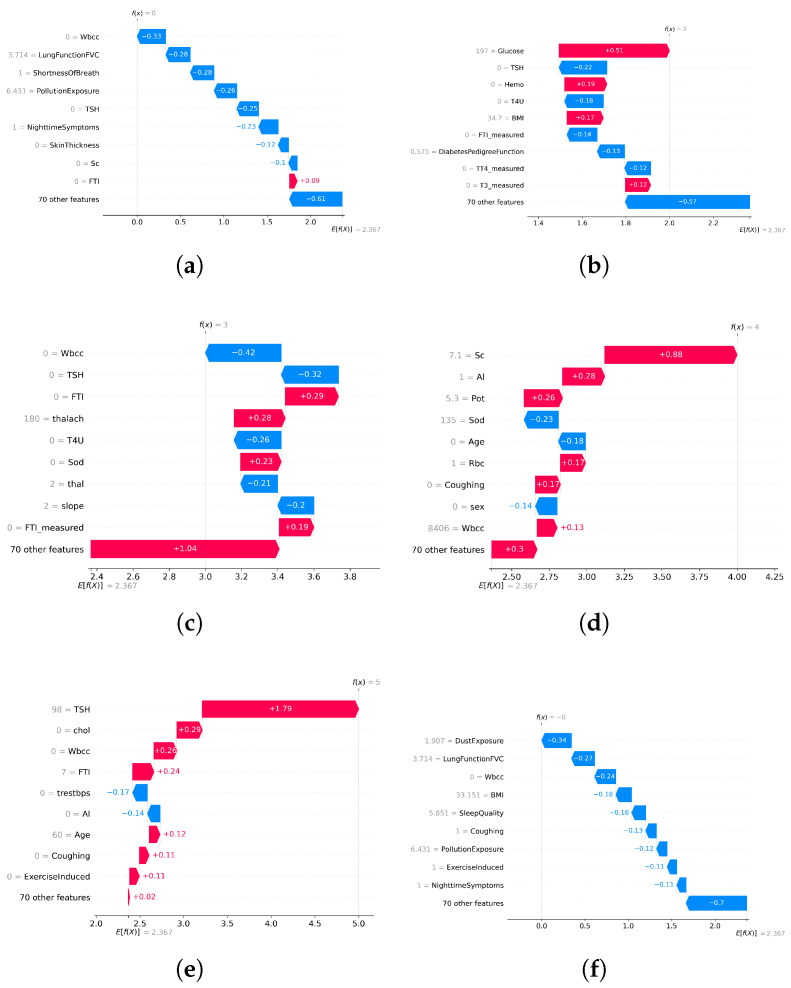
SHAP waterfall plots of local feature contributions across disease categories; red increases risk, blue decreases. (**a**) Asthma; (**b**) diabetes; (**c**) heart problem; (**d**) chronic kidney disease; (**e**) thyroid problems; and (**f**) combined analysis.

**Figure 7:**
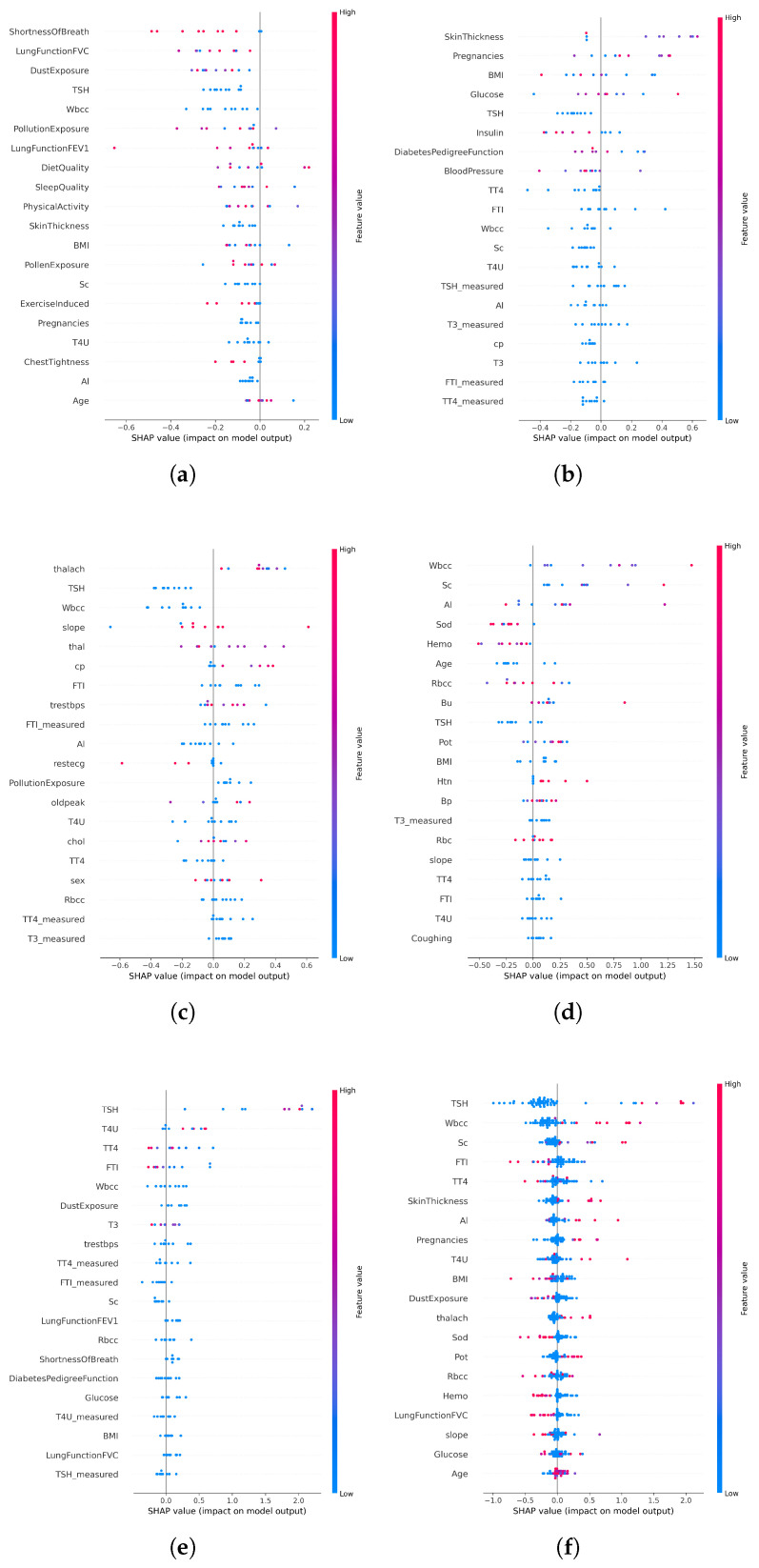
Global SHAP feature importance for the CNN–LSTM model across disease categories. Panels (**a**–**e**) show asthma, diabetes, heart disease, chronic kidney disease, and thyroid disorders; panel (**f**) shows the combined analysis.

In disease-specific settings, SHAP values highlighted distinct clinical markers aligned with established medical knowledge:

- **Asthma:** Respiratory indicators such as white blood cell count and lung function (FVC) emerged as the top contributors (Figure 6a and Figure 7a).
- **Diabetes:** Metabolic and hereditary indicators such as glucose, BMI, and diabetes pedigree function dominated the predictions (Figure 6b and Figure 7b).
- **Heart Disease:** Maximum heart rate (thalach), chest pain type, and slope were key predictors (Figure 6c and Figure 7c).
- **Chronic Kidney Disease:** Serum creatinine and potassium stood out as critical features (Figure 6d and Figure 7d).
- **Thyroid Disorders:** Hormonal markers such as TSH and T4U were consistently dominant (Figure 6e and Figure 7e).

When applied to the unified AI-RiskX model, SHAP revealed that the key features identified in individual disease analyses remained highly influential. As shown in Figure 6f and Figure 7f, variables such as TSH, BMI, white blood cell count, and dust exposure retained high contribution scores, confirming their consistent relevance across both the individual and integrated contexts.

This comparative explainability analysis confirms that AI-RiskX preserves medically meaningful feature relationships, even in a multidisease setting. Contrary to concerns that data fusion might blur condition-specific signals, our findings demonstrate that the model maintains and, in some cases, enhances the discriminative power of critical clinical indicators. By applying SHAP post hoc to the fused representation, AI-RiskX delivers both high predictive performance and clinically interpretable insights. This not only improves trust and accountability but also supports robust, transparent clinical decision-making across diverse conditions.

### 4.7. Implementation

To enhance the clinical usability and real-world deployment, the AI-RiskX approach was implemented through a web-based interactive interface developed using the Gradio library [56]. This interface enables seamless interaction between healthcare professionals and the predictive model, offering an intuitive environment for patient data entry, automated disease risk prediction, and real-time interpretability.

Unlike conventional black-box AI systems, AI-RiskX integrates interpretable deep learning with clinically informed rule-based logic to ensure transparency, trust, and alignment with healthcare decision-making processes.

The interface is structured into four main functional components:

#### 4.7.1. Data Entry Form

The interface begins with a structured, user-friendly data entry panel that allows clinicians to input patient-specific demographic and clinical information for multidisease risk assessment. Standardized fields are provided for essential attributes such as age (via slider), sex (radio buttons), pregnancy status, and ethnicity. Disease-specific parameters, such as blood pressure, glucose levels, and organ-specific indicators, can be entered through dedicated tabs tailored to cardiac, diabetes, thyroid, respiratory, and kidney conditions.

**Figure 8:**
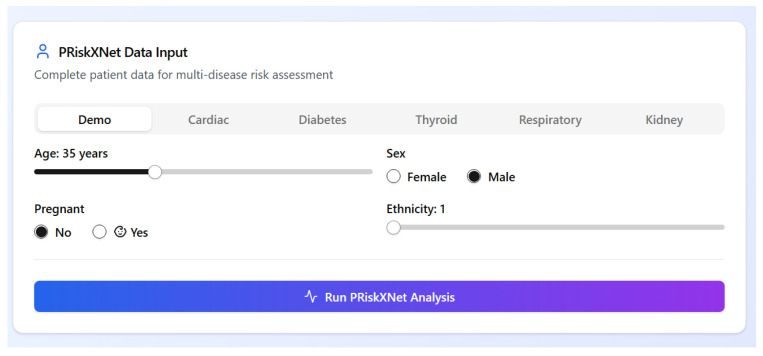
Demographic and clinical information data entry panel.

This modular design supports both general and condition-specific evaluations, while the clean layout ensures accessibility, even for users with minimal technical training. An overview of the data input panel is presented in Figure 8.

#### 4.7.2. Disease Risk Prediction and Rule-Based Stratification

Once the patient’s data are submitted, the AI-RiskX system powered by a hybrid CNN–LSTM architecture predicts the most probable disease class. The output corresponds to one of six categories: heart disease, diabetes, asthma, thyroid disorder, chronic kidney disease, or no disease.

In parallel, a demographic-aware, rule-based stratification module categorizes patients into three risk levels: *high risk*, *at risk*, or *not at risk*. This stratification is determined by combining the predicted disease with critical demographic features such as age group (e.g., elderly or pediatric) and pregnancy status. For instance, an elderly patient (age ≥ 60 years) diagnosed with thyroid disorder would be flagged as *high risk*, with an explanatory note referencing their increased vulnerability and associated mortality risk.

**Figure 9:**
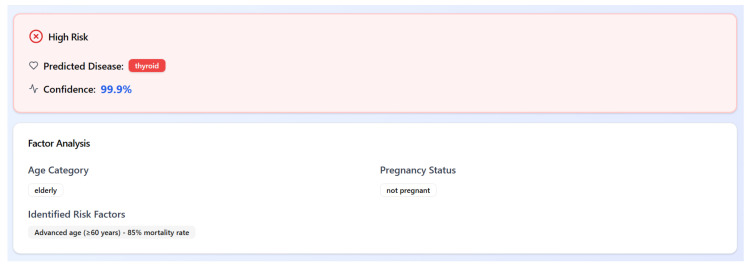
Example output showing predicted disease, confidence, risk level, and demographic reasoning.

This hybrid approach combines data-driven predictions with domain knowledge, enhancing medical interpretability and enabling clinicians to act with greater confidence. An example of the system output is shown in Figure 9.

#### 4.7.3. Explainability Through SHAP

To ensure transparency in decision-making, AI-RiskX integrates SHAP (SHapley Additive Explanations) [34], a widely adopted method for model interpretability. SHAP quantifies the contribution of each input feature to the model’s prediction, offering both local (individual-level) and global (population-level) insights. These were visualized using bar plots and summary charts integrated into the interface.

**Figure 10:**
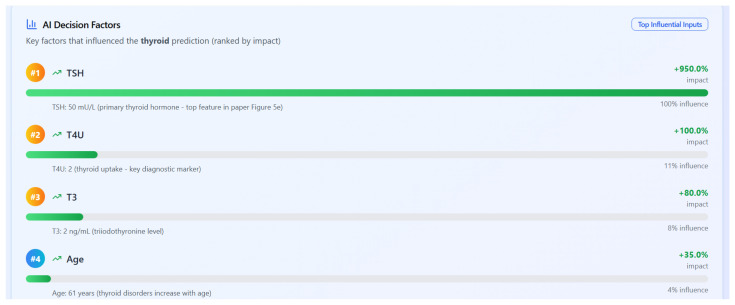
SHAP-based feature importance ranking for thyroid prediction (TSH as the most influential feature, T4U, T3, and age).

Figure 10 presents a ranked list of influential features for a thyroid disorder prediction, as computed by the SHAP values. Thyroid-stimulating hormone (TSH) has the highest impact, followed by T4U (thyroid uptake), T3 (triiodothyronine), and patient age. These results align with clinical expectations and support explainable AI in a diagnostic context.

## 5. Discussion and Conclusions

We introduced **AI-RiskX**, an interpretable hybrid CNN–LSTM framework for multidisease risk prediction, combining SHAP-based explanations with a demographic-aware rule-based module. AI-RiskX classifies patients already diagnosed with COVID-19 or related infections, supporting timely intervention and resource allocation. Trained on a harmonized dataset covering asthma, diabetes, heart disease, chronic kidney disease, and thyroid disorders, AI-RiskX achieved 98.78% accuracy, outperforming LSTM, BiLSTM, CNN, and LLM–BERT baselines. SHAP confirmed medically relevant features, while the rule-based module stratified patients into *high-risk*, *at-risk*, and *not-at-risk* categories.

Extensive cross-validation and statistical testing confirmed the robustness and reliability of AI-RiskX, demonstrating consistently high predictive performance across all folds.

AI-RiskX supports rapid prioritization of high-risk patients in pandemic settings, guiding ICU, ventilation, and treatment allocation. Explainable predictions foster clinician trust, and integration hospital systems enables near real-time updates.

By unifying five heterogeneous datasets, modeling comorbidities, and combining CNN–LSTM features with demographic risk indicators, AI-RiskX delivers clinically interpretable risk categories while maintaining fairness and transparency.

In conclusion, AI-RiskX offers accurate, interpretable multidisease risk assessment, addressing the scalability, fairness, and trust critical in pandemics. The current study is limited by the absence of external validation and possible differences between synthetic and real-world data. Future work will assess AI-RiskX on independent cohorts to verify its robustness and applicability in clinical settings.

## Figures and Tables

**Table 1:** COVID-19 key risk factors and mortality rates.

Risk Factor	Mortality/Severity Rate (%)
Heart Disease	52.0
Chronic Kidney Disease	48.0
Hypertension	28.0
Diabetes	24.0
Severe Asthma (ICU)	23.3
Elderly (≥60 years)	85.0
Children Severe COVID-19 Cases	30.0
Children ICU Admissions	28.0
Children Invasive Ventilation	6.0
Pregnancy-Related Severe COVID-19	10.2

**Table 2:** Related work discussion.

Authors	Disease Scope	AI Methodology	Explainability Used	Vulnerable Groups	Limitations
Bottrighi et al. [24]	Single (COVID-19)	JRIP (rule-based ML)	None	No	Class imbalance, no explainability
Zhao et al. [36]	Single (Immune Disorders)	XGBoost (VDJMiner)	Yes (SHAP)	No	Limited to immune repertoire data
Quiroz et al. [25]	Single (COVID-19)	kNN, SVM, NN	None	No	Not generalizable
Duckworth et al. [35]	Single (COVID-19-related Risks)	NN + SHAP	Yes (SHAP)	No	Single-disease, limited dataset sources
Tasin et al. [37]	Single (Diabetes)	XGBoost, SMOTE	Yes (LIME, SHAP)	No	Small dataset, lacks demographic profiling
El-Sofany et al. [17]	Single (Diabetes)	XGBoost + SMOTE	None	No	Single-disease, no explainability
Srinivasu et al. [31]	Single (Diabetes)	CNN + BiLSTM	None	No	Image-based, no demographic analysis
Guleria et al. [18]	Single (Heart)	SVM + SHAP/LIME	Yes (SHAP, LIME)	No	Small dataset, no deep learning
Yilmaz et al. [19]	Single (Heart)	RF, LR, SVM	None	No	No explainability, no vulnerable group identification
Prathibha et al. [20]	Single (Thyroid)	ResNet (DL)	None	No	Only image-based data, single disease
Zhang et al. [21]	Single (Thyroid)	Xception CNN	None	No	Focused only on ultrasound/CT
Awal et al. [22]	Single (Asthma)	BOMLA (ML)	None	No	Dataset imbalance, no explanation logic
Heris et al. [23]	Single (Asthma)	KNN	None	No	Risk of overfitting, small dataset
Ahmed et al. [26]	Partial (CVDs)	ML + statistical modeling	None	No	Genomic focus, lacks clinical variables
**AI-RiskX (Ours)**	**Multi (5 diseases)**	**CNN-LSTM + Rule-based + SHAP**	**Yes (SHAP)**	**Yes (children, elderly, pregnant)**	**None: unified dataset, deep + rule fusion, explainability included**

**Table 3:** AI-RiskX dataset overview.

Property	Description
Total samples	26,125
Number of features	79
Feature categories	Demographics, Lifestyle, Clinical measurements, Thyroid tests, Cardiac, Respiratory, Allergy/Immunology
Target variable	Multi-class disease label
Source integration	Five heterogeneous datasets (Asthma, Diabetes, Heart, Kidney, Thyroid)

**Table 4:** Feature group overview.

Group	Count	Examples
Demographic	5	Age, Sex, Ethnicity
Lifestyle	5	Smoking, Physical Activity
Cardiac	9	Trestbps, Chol, Thalach
Diabetes	6	Glucose, Insulin, BMI
Kidney	11	Bu, Sc, Sod
Thyroid	16	TSH, TT4, T3
Asthma	15	Wheezing, Coughing

**Table 5:** Class distribution in the training set after SMOTE oversampling (applied only to the training subset).

Label	Disease	Samples	After SMOTE
0	No disease	22,420	22,420
1	Asthma	124	22,420
2	Diabetes	952	22,420
3	Heart	526	22,420
4	Kidney	250	22,420
5	Thyroid	1853	22,420
**Total**		**26,125**	**134,520**

**Table 6:** Summary of the proposed CNN–LSTM architecture for AI-RiskX.

Stage	Layer(s)	Output Shape
Input	79 numeric clinical features	(79, 1)
Feature Extraction	3× Conv1D (64 filters, k = 3/5/7, ReLU) + BatchNorm	(79, 64) each
Feature Enrichment	2nd Conv1D set (same configuration)	(79, 64) each
Pooling	MaxPooling1D (pool size = 2) for each conv branch	(39, 64) each
LSTM Branch	LSTM(64) + BatchNorm → LSTM(32, no recurrence)	(32)
Fusion	Flatten + Concatenate all branches	(vector)
Dense Layers	Dense(64, ReLU) → Dense(32, ReLU) + Dropout(0.3)	(32)
Output Layer	Dense(6, softmax)	(6)

**Table 7:** Performance comparison between classical and deep learning models for multidisease prediction under different dataset configurations.

Approach	Dataset	Model	Accuracy (%)	Precision (%)	Recall (%)	F1-Score (%)
Standalone	Heart	LSTM	96.56	96.56	96.55	96.55
	Diabetes	LSTM	96.93	96.93	96.92	96.92
	HHT	LSTM	96.34	96.34	96.33	96.33
	**AD**	**LSTM**	**95.20**	**95.20**	**95.19**	**95.19**
	CKD	LSTM	96.25	96.25	96.24	96.24
	**Heart**	**BiLSTM**	**97.07**	**97.07**	**97.05**	**97.05**
	**Diabetes**	**BiLSTM**	**97.40**	**97.40**	**97.39**	**97.39**
	**HHT**	**BiLSTM**	**96.57**	**96.57**	**96.55**	**96.55**
	AD	BiLSTM	94.78	94.77	94.75	94.75
	**CKD**	**BiLSTM**	**97.50**	**97.50**	**97.49**	**97.49**
	Heart	CNN	89.75	89.75	89.70	89.72
	Diabetes	CNN	86.82	86.82	86.81	86.81
	HHT	CNN	91.21	91.21	91.20	91.20
	AD	CNN	93.32	93.32	93.32	93.32
	CKD	CNN	76.25	76.25	76.23	76.23
Combined	AI-RiskX	Logistic Regression	89.95	88.64	89.95	87.75
	AI-RiskX	Random Forest	96.77	96.34	96.77	96.55
	AI-RiskX	XGBoost	96.99	96.62	96.99	96.80
	AI-RiskX	LLM-BERT	79.35	79.30	79.21	79.26
	AI-RiskX	LSTM	93.16	93.16	93.12	93.13
	AI-RiskX	BiLSTM	90.91	90.91	90.89	90.89
	AI-RiskX	CNN	94.26	94.26	94.24	94.24
	AI-RiskX	CNN-LSTM	94.55	94.55	94.53	94.53
**Combined–Balanced**	AI-RiskX	Logistic Regression	70.68	89.40	70.68	76.21
	AI-RiskX	Random Forest	96.84	96.47	96.84	96.65
	AI-RiskX	XGBoost	96.98	96.68	96.98	96.81
	AI-RiskX	LLM-BERT	86.02	86.01	85.95	85.97
	AI-RiskX	LSTM	96.97	96.97	96.95	96.95
	AI-RiskX	BiLSTM	96.13	96.13	96.12	96.12
	AI-RiskX	CNN	98.67	98.67	98.66	98.66
	AI-RiskX	CNN-LSTM	98.78	98.79	98.77	98.77

**Table 8:** Ablation study results comparing different architectural configurations on the balanced dataset.

Model	Accuracy	Precision	Recall	F1-Score
LSTM only	96.97	96.97	96.95	96.95
CNN only	98.67	98.67	98.66	98.66
**CNN–LSTM (AI-RiskX)**	**98.78**	**98.79**	**98.77**	**98.77**

**Table 9:** Statistical summary of cross-validation results (Mean ± Standard Deviation).

Metric	Mean ± Std (%)
Accuracy	98.72 ± 0.11
Precision	98.71 ± 0.10
Recall	98.73 ± 0.09
F1-score	98.71 ± 0.08
AUC	99.52 ± 0.03

## Data Availability

The data supporting the findings of this study are available from the corresponding author upon reasonable request.
